# Is the ischemia found in normal pressure hydrocephalus secondary to venous compression or arterial constriction? A comment on Ohmura et al

**DOI:** 10.1186/s12987-025-00640-1

**Published:** 2025-03-19

**Authors:** Grant Alexander Bateman, Alexander Robert Bateman

**Affiliations:** 1 Department of Medical Imaging, John Hunter Hospital, Locked Bag 1 Newcastle Region Mail Center, Newcastle, NSW 2310 Australia; 2 School of Medicine and Public Health, College of Health, Medicine and Wellbeing, Newcastle University, Callaghan Campus, Newcastle, NSW Australia; 3 College of Engineering, Science and Environment, Newcastle University School of Engineering, Callaghan Campus, Newcastle, NSW Australia

**Keywords:** Normal pressure hydrocephalus, Cerebral blood volume, Cerebral blood flow, Ischemia

## Abstract

**Background:**

In a recent study of normal pressure hydrocephalus published by Ohmura et al., there was a progressive reduction in the cerebral blood volume within the cortex, as measured by near-infrared spectroscopy, following an increase in the intracranial pressure from an infusion study. The authors contend that this reduction in blood volume occurred due to the collapse of the venous structures, starting from the smallest veins adjacent to the capillaries and involving the entire venous outflow tract. We wish to outline some problems with this interpretation.

**Main body:**

It has been previously shown that venous collapse secondary to an increase in intracranial pressure always starts at the most distal point in the veins. The critical buckling pressure for a tube depends on the cube of the ratio of the wall thickness and the internal diameter. The smallest veins have ratios which are larger than the distal cortical veins, so the latter are the ones to collapse first. The collapse of the distal venous outflow cuff always leads to an increase in the transmural pressure of the veins upstream from it, leading to venous dilatation and not a reduction in venous volume. Only a simultaneous arteriolar constriction of a greater volume than the venous volume increase can account for the progressive reduction in blood volume, which occurs once the ICP is greater than the sinus outflow pressure in normal pressure hydrocephalus.

**Conclusions:**

The reduction in cerebral blood volume which occurs in the cortex in normal pressure hydrocephalus cannot be due to widespread venous collapse. Therefore, there must be a large component of arteriolar constriction accompanying this disease.

## Background

We read with interest a recent study published by Ohmura et al. [1]. In this study, the authors performed a controlled cerebrospinal fluid (CSF) infusion study in patients with normal pressure hydrocephalus (NPH), and compared the intracranial pressure (ICP) to different intracranial hemoglobin species using near-infrared spectroscopy. The hemoglobin types of most interest to the current discussion are the total hemoglobin, which reflects the cerebral blood volume (CBV) and the deoxyhemoglobin, which reflects the oxygen usage of the cortex [1]. Both were measured in the outer few millimeters of the cortex by using near-infrared spectroscopy. As the infusion progressed from an ICP of -20 mmHg to + 30mmHg (with reference to the external auditory meatus), the total hemoglobin decreased throughout the infusion process, indicating a steady reduction in the CBV, but the deoxyhemoglobin initially decreased and then increased upon reaching 10.8 mmHg, indicating an increase in oxygen extraction fraction and therefore the beginning of ischemia from this point on [1]. The findings suggested cerebral blood flow impairment begins at a very low intracranial pressure in NPH, indicating that cerebral auto regulation is not functioning properly [1]. The authors contend that with the ischemia developing at such a low ICP in NPH, there must be a total collapse of the venous outflow, starting from the smallest veins and extending toward the largest, with a quadratic increase in the outflow resistance being behind the reduced blood flow. We wish to discuss this interpretation of the results from a hydraulic engineering standpoint to see if this scenario is feasible.

## Main text

When Ohmura et al. took the ICP from − 20 mmHg to + 30 mmHg as part of their study, we suggest there were two distinct phases. The authors calculated the mean intraparenchymal venous pressure to be 8.3 mmHg at the maximal compliance of the system, as this was the ICP when the maximal compliance occurred. They contend that, theoretically, the intracranial compliance should be maximal when the venous transmural pressure (internal pressure minus external pressure) is zero [1]. We suggest phase 1 of the test represents that portion of the infusion study, where the ICP was increased from − 20 mmHg to 8.3 mmHg and phase 2 from this figure to 30 mmHg. In phase 1 the reduction in CBV, as noted by Ohmura et al., would be entirely due to a reduction in the size of the veins. This is because the venous transmural pressure would be highly positive initially (i.e. a very low ICP compared to the mean vein pressure), therefore the veins would be circular and increased in volume due to the elastic nature of their walls [2]. As the transmural pressure was decreased with a progressive increase in the ICP, the vein volume would decrease due to the elastic wall relaxation [2]. This occurs until the ICP reaches the mean cerebral venous pressure and then some collapse of a portion of the venous outflow will occur, but which portion?

### The distal veins collapse first

In the second phase of the infusion study (i.e., where the ICP increases from 8.3 mmHg to 30 mmHg), Ohmura et al. contend that the collapse starts in the smallest veins. This is despite the author’s citing Cirovic et al. [3], who state the venous collapse should begin at the most distal point in the vascular system, where the venous pressure is lowest in comparison to the ICP (for blood to flow along the vascular tree, the pressure has to be lowest in the distal veins compared to upstream). Cirovic et al., also indicate that to empty the veins completely requires the ICP to be above the arterial inflow pressure [3], which never occurs in an infusion study. Ohmura et al. then attempt to refute Cirovic et al.’s findings by suggesting that the proximal veins adjacent to the capillary are the actual beginning of the collapse because these proximal veins have thinner walls and are therefore more susceptible to compression [1]. This statement is in error because it fails to take into account all of the variables that govern the collapse of tubes. The site of the start of venous collapse depends on the critical buckling pressure, which depends on more than just the wall thickness. The critical transmural pressure at which buckling of a collapsible tube begins depends on the stiffness of the wall (Young’s elastic modulus), Poisson’s ratio (how the wall deforms under a tensile load), the wall thickness, the internal diameter of the vessel and the vessel length [4]. Of these, by far and away the most important is the ratio of the wall thickness to the internal diameter because the critical buckling pressure is proportional to the cube of this ratio. The buckling pressure is mostly related linearly to the other variables [4]. If the buckling pressure were dependent only on the wall thickness, then the capillaries would be the ones to collapse first because they are the vessels with the thinnest walls. The average internal diameter of a human cerebral capillary is 8 μm and the wall thickness is 1 μm [5], giving a wall thickness to internal diameter ratio of 0.13. We are unaware of similar measurements being available for the cerebral venules in humans, but in dogs this ratio is similar being 0.14 [6]. In humans the average cortical vein adjacent to the sagittal sinus has an internal diameter of 3.3 mm and has a wall thickness of 0.044 mm [7], giving a ratio of 0.013 (tenfold less). The cube of the ratio of thickness divided by diameter for capillaries compared to veins is therefore 1000. The theoretical elastic modulus for capillaries is 0.68 MPa [5] and the current authors calculated this metric for cortical veins to be 0.16 MPa [7], indicating the capillary walls are stiffer than the veins. The Poisson’s ratio for the tensile deformation in most materials varies between 0 and 0.5, with a likely value for most blood vessels being 0.4 [5]. The capillaries are shorter than the cortical veins, making them harder to collapse from this viewpoint. Given the critical buckling pressure is proportional to the cube of the thickness to diameter ratio, and the capillaries are stiffer and shorter than veins, then the capillaries and smallest veins will have a critical buckling pressure that is at least 1000 times greater than the distal cortical veins. This buckling pressure is probably very low in cortical veins, being approximately − 0.1 mmHg in a dog model using the inferior vena cava [2]. The figure for capillaries and venules will be much larger, approximating − 100 mmHg. Therefore, the smallest capacitance vessels do not buckle first, the distal veins do, just adjacent to the sinus wall.

### Vein collapse or dilatation as the ICP increases?

**Fig. 1:**
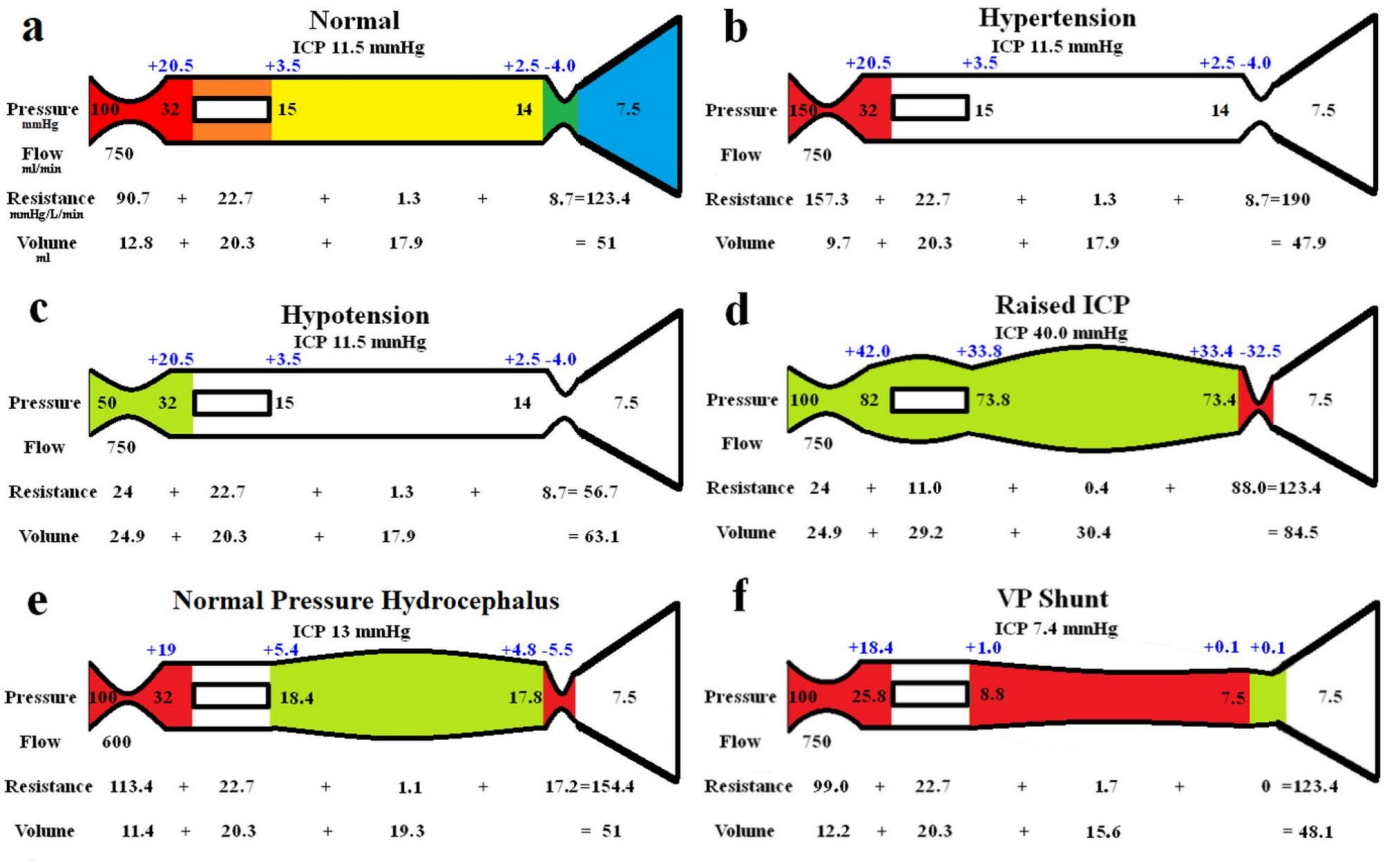
Results of modelling. **a**. Depicts the normal findings. The red segment is the arterial, orange is the capillary, yellow is the veins, green is the outflow cuff and blue is the venous sinus. The vascular pressures are shown within the vessels. The blue numbers are the transmural pressures at each site. The resistances and volumes for each segment are shown below the vessel. **b**. Shows the findings in hypertension with the red area indicating an increase in resistance in the arteries. The perfusion pressure is increased and the CBV is reduced.**c**. Shows the findings in hypotension with the green area highlighting a reduction in resistance in the arteries. The perfusion pressure is reduced and the CBV is increased. **d**. Shows the findings in raised ICP with increased resistance in the outflow cuff and reduced elsewhere. Note the major resistance moves from the arteries to the outflow cuff and a backup of pressure dilates the veins and capillaries. The perfusion pressure is reduced and the CBV is increased. **e**. Shows the global findings in normal pressure hydrocephalus. It should be noted that the changes are a mixture of 1b and 1d combined, hence the CBV is normal. **f.** Shows the findings following shunt insertion with decreased resistance in the outflow cuff. Note the reduction in the arterial resistance as compared to NPH suggesting reversibility. The perfusion pressure is increased and the CBV is reduced

Given that the collapse first occurs at the most distal point of the cortical veins as the transmural pressure becomes negative, how do the upstream veins respond to this? This very topic was modelled recently by the current authors. The current authors developed a lumped parameter study of the global cerebral autoregulation in normal individuals and then applied it to NPH [8]. This model was verified by successfully predicting the cerebral blood volume changes that occurred at the limits of cerebral autoregulation in both animal models and human experiments [8]. A figure from this paper has been reproduced as Fig. 1.

### Reproduced from [8] under a CC BY 4.0 commons licence

The five vascular segments which were modelled are shown in Fig. 1a. The arterial segment in is red, the capillaries in orange, the veins in yellow, the outflow cuff in green and the sinus in blue. The pressures at the beginning and end of each segment were obtained from the literature and are shown within the vessels. The normal blood flow value comes from the literature, with the resistance of each vascular segment being calculated using Ohm’s law and appended below the vessels in Fig. 1. The normal CBV values for each segment and the total CBV were obtained from the literature and are shown below the resistances. The changes in the volume of each segment reflect the inverse square of the change in the resistance as per Poiseuille’s law. The blue numbers represent the transmural pressure gradients between the pressure at the beginning and end of each capacitance vessel segment and the ICP and are obtained by subtraction. Figure 1b-f represent the effects of the differing alterations in the perfusion pressure (arterial pressure – ICP). In these figures, the red segments represent the areas of increased resistance compared to the normal findings and the green sections represent reduced resistance. In Fig. 1b, the upper limit of autoregulation, i.e., an arterial inflow pressure of 150 mmHg, has been modelled. To maintain the normal arterial inflow, the arterial resistance needed to be considerably increased and the total cerebral blood volume was proportionally reduced. Figure 1c models hypotension at the lower limit of autoregulation i.e., 50 mmHg. In order to maintain the normal blood flow rate, the arterial resistance was significantly reduced and an increase in CBV resulted. Figure 1d models the limit of autoregulation with an increased ICP of 40 mmHg. The inflow pressure and flow rate are preserved, so the total resistance across all segments combined must be normal. To maintain a normal blood flow, the arterial resistance was maximally reduced (as per Fig. 1c), with the capillaries and veins being maximally dilated because the arterial pressures were passed further into these segments by the dilated arteries. In order for the normal arterial blood flow to be preserved, the outflow cuff was constricted so that the total resistance reverted back to normal. Note the CBV was greatly increased in this model. Figure 1e models NPH by reducing the blood flow by 20% in NPH and setting the cerebral blood volume to be unchanged at 51 ml. This was because 4 published studies indicated the global CBV was normal in NPH, despite the significant CBF reduction [8]. The outcome was a 73% increase in arterial resistance and a 27% increase in total venous resistance including the cuff value, indicating the arterial resistance predominated as the cause of the reduced blood flow. Figure 1f models the effect of inserting a medium pressure shunt valve in NPH. The literature indicates the arterial flow reverts to normal if patients improve following a shunt [8]. Note the average intraparenchymal venous pressure is 8.2 mmHg in Fig. 1f and this is close to Ohmura et al.’s figure for the venous pressure at maximal compliance i.e., the minimal venous volume and maximal compliance occurs following a shunt. The CBV is low at 48.1 ml.

The outcome of the modelling was to show that any change which increased the cerebral perfusion pressure but maintained the arterial flow rate (i.e., either an increase in arterial inflow pressure as in Fig. 1b, or a reduction in ICP as in Fig. 1f) reduced the cerebral blood volume due to either arterial and or venous constriction. Any change that reduced the cerebral perfusion pressure but maintained the arterial flow rate (i.e., either a decrease in arterial inflow pressure as in Fig. 1c, or an increase in ICP as in Fig. 1d) increased the CBV. Figure 1e shows that any increase in ICP (even a mild increase as in NPH) will increase the venous component of the blood volume. This finding is backed up by animal modelling. Grubb et al. reduced the arterial pressure in Rhesus monkeys to their limit of autoregulation and the CBV increased by 25% compared to the normotensive animals [9]. Note the CBV increased by 24% in Fig. 1c similar to the findings in the monkeys. In the same primate model, increasing the ICP from 8.6 to 71 mmHg increased the CBV by 66%, increasing the ICP further to 94 mmHg did not alter the CBV but did reduce the blood flow [10]. This indicates the autoregulation limit was transgressed at 94 mmHg and the elastic limit for the venous distension was also probably reached. These findings are identical to our findings, which show that raising the ICP to the limit of autoregulation increases the CBV by 66% (see Fig. 1d). Globally, for a total CBV to be normal as per the literature in NPH, there must be an increase in arterial resistance i.e., the brain apparently chooses to be ischemic by constricting rather than dilating its arteries to maintain flow. If the CBV were to continue to reduce within the outer few millimetres of the brain in NPH, as the ICP increases, (as Ohmura et al. have shown), then the cortex arterial volume must decrease at a faster rate than the increase in venous volume (as normally occurs as the ICP increases) indicating a progressively increased arterial resistance with an ICP increase. Therefore, with an increase in ICP above 8.3 mmHg, the brain chooses to progressively make itself even more ischemic. We have discussed why such an outcome may be beneficial to the brain in the short term in NPH in the original paper [8]. Interested readers are directed here for a fuller discussion.

## Conclusions

The findings of Ohmura et al. that the cerebral blood volume progressively decreases as the ICP is increased from a value of 8.3 mmHg to 30 mmHg cannot be due to a progressive total venous collapse because (1) the capillaries and deepest venules are too resistant to buckling for the collapse to start here and (2) when a progressive collapse occurs at the outflow cuff, the upstream veins actually dilate and do not reduce in volume. The only way for Ohmura et al.’s findings to be accommodated is if the arterial tree becomes progressively more constricted as the ICP is increased i.e., the brain chooses to make itself even more ischemic as the ICP increases.

## Data Availability

No datasets were generated or analysed during the current study.

## Abbreviations

CSF: Cerebrospinal fluid
CBV: Cerebral blood volume
ICP: Intracranial pressure
µm: Micrometers
ml: Milliliters
mm: Millimeters
mmHg: Millimeters of mercury
MPa: Megapascals
NPH: Normal pressure hydrocephalus
